# Antipsychotics drug aripiprazole as a lead against breast cancer cell line (MCF-7) *in vitro*

**DOI:** 10.1371/journal.pone.0235676

**Published:** 2020-08-03

**Authors:** Adnan Badran, Atia tul-Wahab, Humaira Zafar, Nayab Mohammad, Rehan Imad, Mariam Ashfaq Khan, Elias Baydoun, M. Iqbal Choudhary

**Affiliations:** 1 Faculty of Pharmacy and Medical Sciences, University of Petra, Amman, Jordan; 2 Dr. Panjwani Center for Molecular Medicine and Drug Research, International Center of Chemical and Biological Sciences, University of Karachi, Karachi, Pakistan; 3 Department of Biology, American University of Beirut, Beirut, Lebanon; 4 H. E. J. Research Institute of Chemistry, International Center for Chemical and Biological Sciences, University of Karachi, Karachi, Pakistan; 5 Department of Chemistry, Faculty of Science and Technology, Universitas Airlangga, Surabaya, Indonesia; Duke University School of Medicine, UNITED STATES

## Abstract

Breast cancer is the second leading cause of death among women globally. The existing treatment options for breast cancer are largely associated with severe toxicities, and lower efficacies. Therefore, there is an urgent need for the development of non-toxic effective drugs against breast cancer. For this purpose, drug repositioning strategy was used to evaluate the anti-cancer potential of a library of heterocyclic drugs. The major advantage of drug repurposing is that the pharmacokinetic, pharmacodynamic, and toxicity profiles of drugs are well documented. In the current study, we screened 97 drugs of different chemical classes, and among them aripiprazole, an antipsychotic drug, was found to be sufficiently active against breast cancer cell line MCF-7. Aripiprazole showed a cytotoxicity (IC_50_ = 12.1 ± 0.40 *μ*M) to MCF-7 cells, comparable to the standard anticancer drug doxorubicin (IC_50_ = 1.25 ± 0.34 *μ*M). Aripiprazole was also found to be active against other cancer cell lines, including MDA-MB-231 (IC_50_ = 19.83 ± 0.27 *μ*M), AU565 (IC_50_ = 18.02 ± 0.44 *μ*M), and BT-474 (IC_50_ = 36.42 ± 0.12 *μ*M). Aripiprazole significantly inhibited the cell cycle progression at subG_0_G_1_ phase, and enhanced apoptosis in MCF-7 breast cancer cells. The drug was also able to significantly increase the nuclear condensation, and modulated the expression of certain genes involved in breast cancer, such as caspases 3, and 9, BAK-1, C-MYC, BCL2L1, BCL-10, and BCL-2. Further studies are needed to explore the effect of aripiprazole on intrinsic and extrinsic pathways of apoptosis in cancer cells.

## Introduction

According to a report of the American Cancer Society, over 0.253 million new cases of invasive breast cancer were diagnosed during 2017–18 among women, and 2,470 cases in men [1]. Approximately one-third of all breast cancers, and two-thirds of postmenopausal breast cancers are hormone-dependent (estrogen-dependent) cancers. Breast cancer is subdivided into three types based on receptors, including progesterone receptor (PR), estrogen receptor (ER), and the human epidermal growth factor receptor 2 (HER2, also known as ErbB2). Majority of the breast cancers are ER+, followed by triple negative breast cancers (TNBC; 15–20% of breast cancers), and HER2 (10–15% of breast cancers).

Aripiprazole has a unique pharmacological profile as it can act as serotonin 5-HT1A, and partial dopamine D2 agonists, and 5-HT2A antagonist. The action of aripiprazole on D2 receptor is dopamine-dependent, *i*.*e*. it acts as functional antagonist under hyperdopaminergic conditions, while exhibiting functional agonist properties under hypodopaminergic conditions [2].

The existing treatment options for breast cancers include radio-, chemo-, and hormonal therapies, and surgery, while the advanced form of breast cancer still has no effective treatment [3]. Similarly, existing chemotherapies are associated with severe toxicities, which limits their use in cancer treatments. Therefore, there is an unmet need for the development of non-toxic and effective anti-cancer drugs for breast cancer treatment [4].

In the present study, drug repositioning approach was employed to identify the new anticancer uses of existing drugs. Repurposing approach is attracting major scientific interest, in comparison to traditionally used methods of drug discovery [5]. The major advantages of drug repositioning approach are the availability of pharmacokinetic, and pharmacodynamics data, and toxicity profiles, and thus time and the cost required for drug development can be reduced significantly [6]. Successful examples of repositioned drugs include pirlindol for the treatment of multiple sclerosis, which was initially approved for the treatment of chronic depression, and psychotic disorders. Thalidomide is another drug currently used for the treatment of multiple myeloma, which was originally developed as antiemetic agent, metformin was used initially for type 2 diabetes, but is also found to be effective against many cancers. Aspirin was initially developed as an analgesic and antipyretic agent, but now it is used for colorectal cancer, cardiovascular diseases, *etc* [7–9].

Present drug screening project is a systematic attempt to reposition different drugs for their anti-cancer potential on MCF-7 breast cancer cell line. It is a stable epithelioid cell line established at the Michigan Cancer Foundation in 1973, derived by pleural effusion from a breast cancer patient. MCF-7 is estrogen receptor (ER) positive cancer cell lines, used as cellular model for drug lead discovery [10, 11]. Compounds with growth inhibitory potential against MCF-7 can serve as leads for the development, and designing of novel breast cancer drugs.

During the current study, several marketed drugs were evaluated for their potential activity against breast cancer cell line. Among them, antipsychotic drug aripiprazole showed an excellent inhibition of proliferation of MCF-7 cells. The drug was further screened against other breast cancer cell lines including MDA-MB-231 (triple negative breast cancer cells), AU565 (Her2 positive breast cancer cells), and BT-474 (triple positive breast cancer cells). Aripiprazole showed a significant inhibition of all these breast cancer cells. Its mechanism of action was also studied. Aripiprazole (Abilify) is an anti-psychotic drug, developed to treat schizophrenia and bipolar disorders [12, 13]. This is the first report of its anti-cancer effect against breast cancer cell line MCF-7 *in vitro*.

## Materials and methods

Human breast cancer cell line MCF-7 was obtained from the American Type Culture Collection (ATCC HTB22™), USA, Dulbecco's modified eagle medium (DMEM), phosphate buffer saline, 0.25% trypsin EDTA, fetal bovine serum, 3-(4,5-dimethylthiazole-2-yl)-2,5-diphenyltetrazolium bromide (MTT), and doxorubicin were purchased from Sigma Aldrich, USA. Dimethylsulphoxide (DMSO) was purchased from Calbiochem, Germany. Penicillin-streptomycin, and 0.4% trypan blue were purchased from Thermo Fisher Scientific, Germany. Propidium iodide (Biosera, France), paraformaldehyde (Serva, Germany), 4',6-diamidino-2-phenylindole (DAPI) (Invitrogen), annexin V-FITC (Invitrogen), apoptosis detection kit (Thermo Scientific), RNase A (Penicon), cDNA synthesis kit (Thermo Scientific, Catalog No. K1622), DNase I treatment kit (Thermo scientific Catalog No. EN0521), Real time qPCR master mix (Thermo scientific Catalog No. K0221), and primers for selected gene A (Macrogen, Inc., South Korea) were also purchased. RNA was isolated using Trizol Thermoscientific Catalogue No. 15596–026). Antipsychotic drugs, aripiprazole, levosulpiride, and ziprasidone hydrochloride were obtained as gifts from Scilife Pharma (Pvt.) Ltd., and Werrick Pharmaceuticals (Pvt.) Ltd., respectively. Annexin V-FITC contained fluorescein isothiocyanate as fluorochrome. Flow cytometry data collection and analysis were performed on BD FACSCalibur and BD CellQuest Pro, respectively.

### Protocol of the MTT assay

It is a colorimetric assay that measures the reduction of MTT by mitochondrial enzyme *i*.*e*. succinate dehydrogenase. The MTT enters into the mitochondria of cell, where it is reduced to an insoluble formazan salt. The extent of MTT reduction is measured at 570 nm using a micro-plate reader (Spectra Max plus, Molecular Devices, USA). As reduction of MTT can only occur in metabolically active cells, the level of activity is actually a measure of the viability of the cells [14, 15].

Human breast cancer cell line (MCF-7) was cultured in DMEM, supplemented with 5% of FBS, 100 IU/mL of penicillin and 100 μg/mL of streptomycin, and kept at 37 ^o^C in 5% CO_2_ incubator. For the preparation of cell culture, 100 μL/well of cell solution (10 x10^4^ cells/mL) was added into 96-well plate. The plate was incubated overnight, and fresh medium was added after the removal of old medium. The drugs were added in different concentrations into the plate, and plate was again incubated for 48 hrs. After the completion of this incubation, 200 μL MTT (0.5 mg/mL) was added, and plate was incubated for 4 hrs. After this final incubation, 100 μL of DMSO was added to each well to solubilize formazan crystal. The level of MTT reduction to formazan was evaluated by change in absorbance at 570 nm using a micro-plate reader (Spectra Max plus, Molecular Devices, USA). The anti-proliferative activity was recorded as concentration of the inhibitor causing 50% growth inhibition (Eq 1) (IC_50_) of MCF-7 cell line. Doxorubicin was used as a standard drug in this assay.

(Equation-1):

$$\%\;Inhibition=100-\left(\frac{Absorbance\;of\;Test\;Compound-Absorbance\;of\;Blank}{Absorbance\;of\;Control-Absorbance\;of\;Blank}\right)\times 100$$ (1)

### Cell viability assay through fluorescence activated cell sorting (FACS)

For the validation of MTT results, cell viability assay was performed using propidium iodide (PI). For this purpose, cells were seeded in 24-well plate at a concentration of 0.1–0.2 million cells per well, and incubated for 24 hours. On the next day, the cells in triplicate wells were treated with test drugs, and the plates were incubated for 48 hours. After completion of the incubation time, supernatant was collected, and centrifuged at 2,500 rpm at 4 ºC for 10 min. The remaining attached cells were washed with PBS, trypsinized, and centrifuged at 2,500 rpm at 4ºC for 10 min. Pellet of the supernatant and adherent cells were pooled, and washed twice with 500 μL PBS, and centrifuged again. After washing, the cells were resuspended in 200 μL of PI buffer (10X buffer = 0.1 M HEPES, 25 mM CaCl_2_ NaCl 1.4 M dissolved in 1X PBS), and immediately placed in ice. The PI solution (0.01 μg/200 μL) was added one min before the FACS analysis.

### Investigation of apoptosis

#### Apoptosis analysis by flow cytometry

Cells were seeded in 24-well plate at a concentration of 0.2 million cells per well, and allowed to attach by 24-hour incubation. The treatment with the test compounds was carried out for 48 hours and the cells were harvested by trypsinization, followed by washing with PBS twice to remove the media traces by centrifugation at 2,500 rpm at 4 ºC for 10 min each. The cell pellet was resuspended in 500 μL of the annexin V-FITC/ PI buffer (10X buffer = 0.1 M HEPES, 25 mM CaCl_2_ NaCl 1.4 M dissolved in 1X PBS), followed by the addition of annexin V-FITC (Cat # A13199, Thermo Fisher Scientific, Waltham, MA USA 02451) and PI (0.2 ng/500 μL of binding buffer) 5 μL, and 1 μL, respectively, into each sample. Samples were incubated for 15 min, and proceeded for apoptosis analysis on flow cytometer. PI with excitation/emission maxima of 493/636 nm, was detected on FL2 channel, while annexin V-FITC with excitation/emission maxima of 494/518 nm, was detected on FL1 channel. Live cells, PI positive cells (dead cells), and annexin V-FITC positive cells (cells were treated with camptothecin) were used as negative controls to calibrate the FL2, and FL1 channels, respectively.

#### Apoptotic analysis by detection of nuclear condensation through microscopy

The effect of aripiprazole on apoptosis was also studied using DAPI staining to observe nuclear condensation. After the 48 hours of treatment with aripiprazole, the cells were washed three times with 1X PBS. Cells were then fixed by treating with 3% paraformaldehyde (1 mL), followed by incubation and washing with PBS. Cells were again incubated with 500 μL of DAPI (0.1 ng/0.5 mL) for 10 min. On a glass slide, 50–100 μL of mounting media was poured and cover slip containing prepared cells was carefully placed on the slide with cells containing surface facing the mounting media. The prepared slides were observed under the microscope (TE-2000, Nikon, Japan) at 20X for morphological changes, as compared to the untreated control cells.

#### Cell cycle analysis

Cell cycle analysis was carried out by seeding one million cells in a 6-well plate. After 48 hours of incubation with the test drugs, supernatant was collected, and centrifuged at 2,500 rpm at 4 ºC for 10 min. The remaining attached cells were trypsinized, and centrifuged at 25,00 rpm at 4 ºC for 10 min. The combined cell pellet was washed twice with 500 μL PBS, and centrifuged again. After washing, the cells were resuspended in 500 μL PBS, and proceeded for the fixation step. In separate Eppendorf tubes, 500 μL of 70% chilled ethanol was taken, and treated cells were added drop-wise while keeping the tubes at a low speed vortex. The fixed samples were stored at 4 ºC for 24 hours before starting the FACS analysis. Cells were then centrifuged again at 300 g for 20 min at 4 ºC, and washed twice using 1 mL of 1X PBS. RNAse A (1500 U/mL) was prepared in 1.12% sodium citrate in 1X PBS. The RNAase A (125 μL) was added to the cell pellet, and incubated at 37 ºC for 2–3 hours. The test cells were treated with 125 μL PI solution (0.05 mg/ mL in 1.12% sodium citrate solution), and incubated for 30 min. The cells were then analyzed by FACS to determine the stage of cell cycle, which was affected by aripiprazole.

### Quantitative real-time PCR

Trizol was used to isolate the total RNA from MCF-7 cell line after 48 hours treatment with aripiprazole. The single stranded cDNA was then synthesized using 1 μg of each RNA sample through reverse transcription by using cDNA synthesis kit. The real-time PCR was performed for the specified genes using forward and reverse primers using ThermoScientific SYBR Green /ROX qPCR master mix, while β-actin and GAPDH were used as control (Table 1). The mRNA expression was measured using the obtained threshold cycle (Ct) values. The target genes expression levels were measured by comparison with the reference genes β-actin, and GAPDH, and the fold change was calculated in gene expression level by 2^-ΔΔCt^ method.

**Table 1:** Gene specific forward and reverse primer sequences for caspase-3, and -9, BAK1, c-Myc, BCL2L1, BCL2, BCL10, and housekeeping reference gene β-actin and GAPDH.

Gene	Forward primer	Reverse primer
Caspase 3	TTTTTCAGAGGGGATCGTTG	CGGCCTCCACTGGTATTTTA
Caspase 9	AGCCACCTGAGTAGCTTGGA	CTGCACTTTGGGAGGCTAAG
BAK1	TGATAACTTGGGGAGGCAAG	GCTGAATCCTGGGGAACATA
C-Myc	TCAAGAGGCGAACACACAAC	TAACTACCTTGGGGGCCTTT
BCL2L1	AGTTCCCTTGGCCTCAGAAT	TCCTTTCTGGGGAAGAGGTT
BCL2	GTCTGGGAATCGATCTGGAA	CATAAGGCAACGATCCCATC
BCL10	TTTCCTCAGTGCATTTGTGC	ATTGGCAGTTTTCCCATCAG
β –Actin	AGAAAATCTGGCACCACACC	GGGGTGTTGAAGGTCTCAAA

#### Statistical analysis

The EZ-Fit enzyme kinetics program (Perrella Scientific Inc., Amherst, USA) was employed to calculate the IC_50_ values. Experiments were run in triplicate and the IC_50_ values are reported as standard error of mean (S.E.M). The statistical analyses were performed by IBM SPSS softwares, ANOVA, and LSD.

## Results

We screened 97 heterocyclic drugs deposited in Drug Bank of the Dr. Panjwani Center for Molecular Medicine and Drug Research (PCMD), including non-steroidal anti-inflammatory drugs (NSAIDs), β-lactam antibiotics, β-blockers, diuretics, anti-depressants, and many other (S1 Table). Among them, aripiprazole, an anti-psychotic drug, was found to be sufficiently active against breast cancer cell line (MCF-7).

### Aripiprazole inhibited the proliferation of breast cancer cells

Among 97 drugs evaluated for their anti-cancer potential against breast cancer MCF-7 cell line, aripiprazole was found to be active (S1 Table).

**Fig 1:**
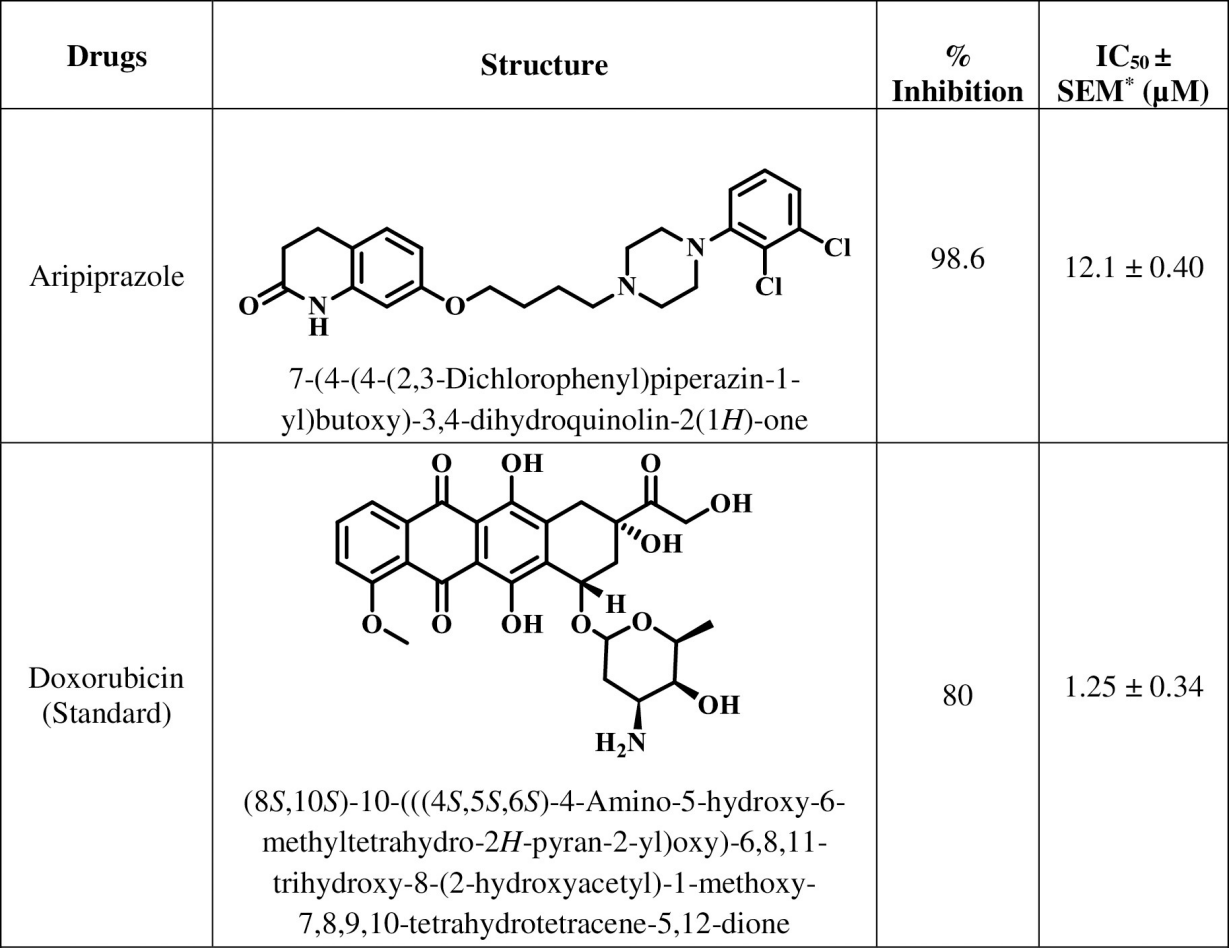
Antipsychotic drug evaluated in the present study.

**Fig 2:**
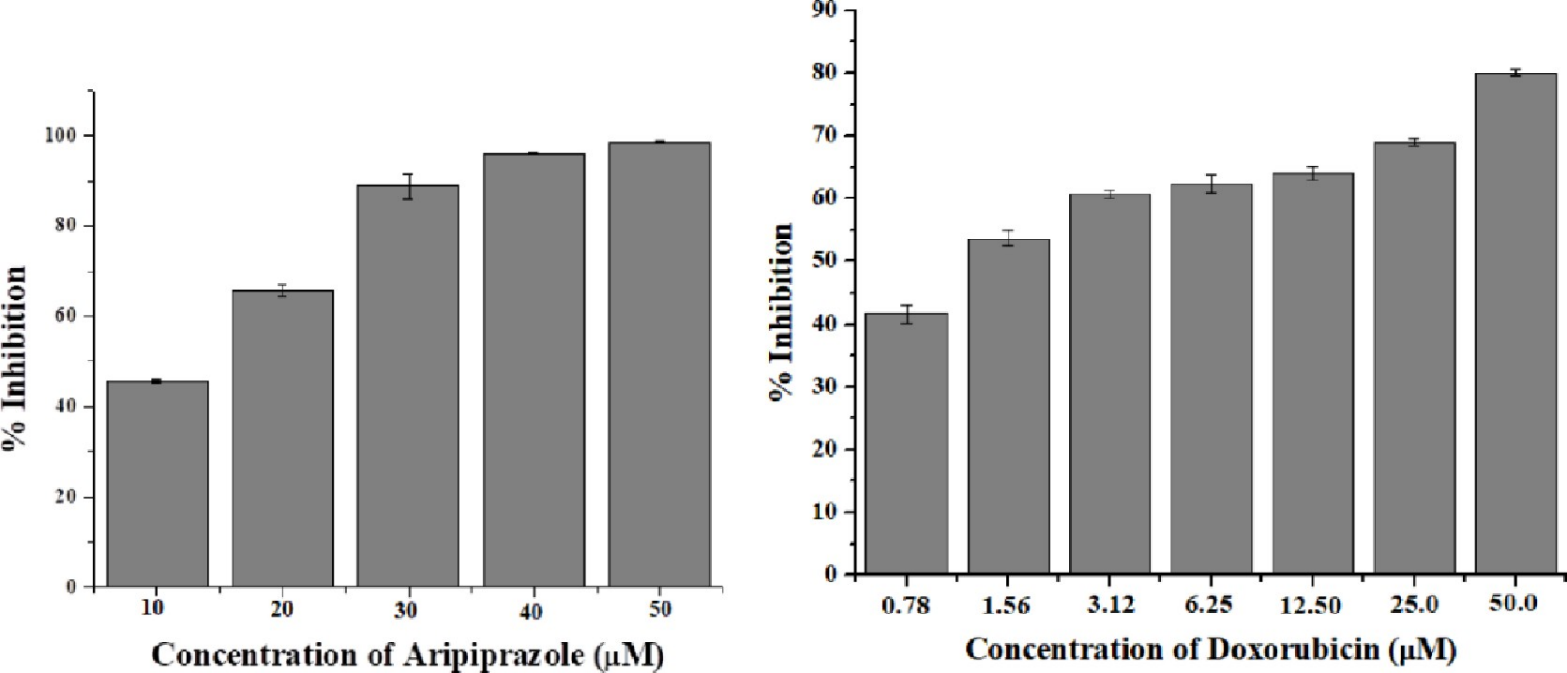
Graphical representation of percent inhibition of MCF-7 cell line. a) Percent inhibition of MCF-7 cells in presence of aripiprazole b) Percent inhibition of MCF-7 cells in presence of doxorubicin (standard inhibitor).

Aripiprazole was able to inhibit the proliferation of breast cancer cells with an IC_50_ value of 12.1 ± 0.40 *μ*M, in comparison to anti-cancer drug doxorubicin (IC_50_ = 1.25 ± 0.34 *μ*M) (Fig 1). Furthermore, the inhibition of growth of MCF-7 cell line by aripiprazole was dose dependent with highest inhibition (90%) at 50 *μ*M (Fig 2). Thus it is a good lead for further modification to become comparable or better to doxorubicin.

**Fig 3:**
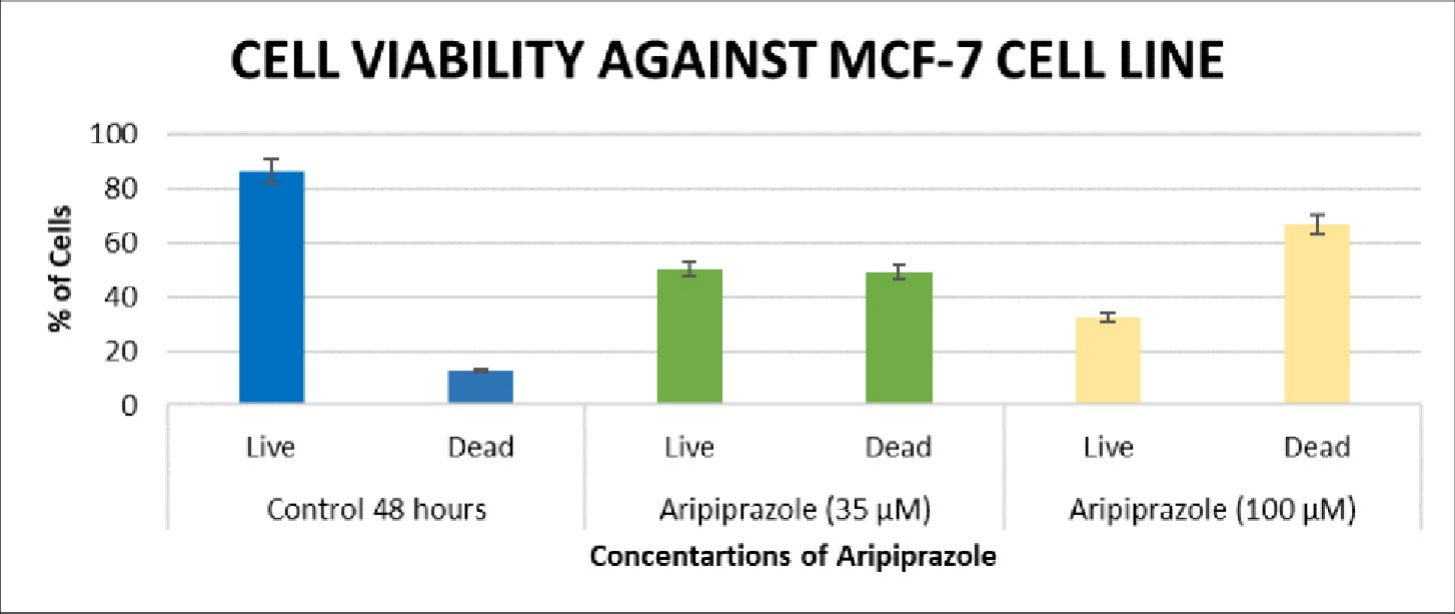
Graphical representation of percent inhibition of MCF 7 cell line in cell viability assay. Percentages of live and dead cells in control group (blue), in presence of aripiprazole 35 μM (green) and 100 μM (yellow).

The results of MTT assay were further validated by performing cell viability assay, based on fluorescence activating cell sorting (FACS) using propidium iodide (PI) [16]. The results of cell viability assay also showed a dose dependency, and found to be in accordance to the results of MTT assay (Fig 3). In the presence of 35 *μ*M of aripiprazole, dead cells were significantly increased to 49.1% (***P<0.001), while dead cells were further increased to 66.7% (***P<0.001) when treated with 100 *μ*M drug. These results were significant as the dead cell percentage was increased from 12.6% in control to 66.7% in aripiprazole treated cells [17, 18].

### Enhanced apoptosis in breast cancer cells

**Fig 4:**
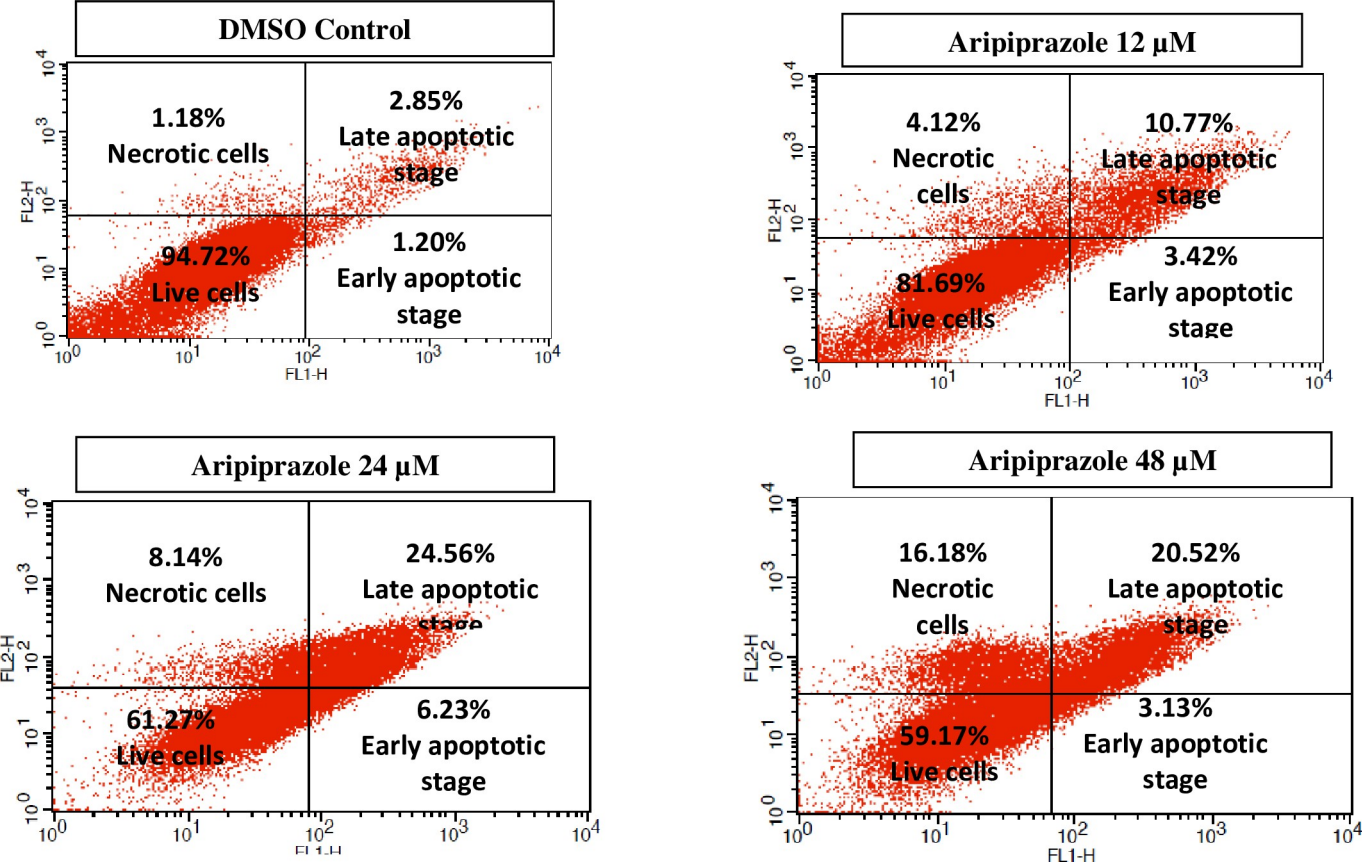
Dot plot representing the total cell count (%). The distribution of viable cells (lower left), dead cells (necrotic) (upper left), early apoptotic (lower right), and late apoptotic cells (upper right) in the four quadrants of dot plots of untreated, aripiprazole treated (12, 24, and 48 μM) MCF-7 cell line. Total 10,000 cells in triplicate were analyzed during sample acquisition.

Apoptosis is the process of programmed cell death that play a key role in physiological and pathological conditions. The increase or decrease in apoptosis can lead to several diseases. For instance, in cancer, decreased apoptosis lead to increase proliferation of cancer cells, those transformed to malignant cells [19]. Therefore, potentiating the process of apoptosis in cancer cells is an important strategy in anti-cancer drug development. Based on the above-mentioned facts, we focused our studies to evaluate the mechanism by which aripiprazole inhibited the cell proliferation. Cell apoptosis assay was performed by staining the cells with annexin V-FITC and PI, through flow cytometry (Fig 4). The presence of only annexin V-FITC positive cells indicated early apoptosis, presence of both annexin V-FITC and PI positive cells indicated late apoptosis, while only PI positive cells indicated necrotic cells.

Cells were treated with aripiprazole at 12, 24, and 48 *μ*M concentrations, and analyzed after 48 hrs using flow cytometry. DMSO was used as a negative control, and it showed 94.72% cells alive, while 1.18% dead (necrotic) cells. Cells (1.20 and 2.85%) were found to be in late and early apoptotic stages, respectively. In contrast, aripiprazole at 12 *μ*M caused a significant increase in apoptosis with 3.42 (*P<0.5) and 10.77% (**P<0.01) in early and late apoptotic stages, respectively, while the necrotic cells were increased to 4.12% (*P<0.5). The increase in concentration of aripiprazole (24 *μ*M) resulted in a significantly enhanced apoptosis with 6.23 (**P<0.01) and 24.56% (***P<0.001) in early and late apoptotic stages, respectively, and necrotic cells were increased to 8.14% (**P<0.01). The number of viable cells was also significantly decreased to 61.27% (***P<0.001) in 24 *μ*M aripiprazole treated cells. The results were dose dependent as the highest concentration of aripiprazole *i*.*e*. 48 μM showed further decrease in live cells upto 59.17% (***P<0.001). While the cell count was highest in necrotic stage (16.18%) (***P<0.001). These results indicated that aripiprazole has inhibited the cell proliferation in MCF-7 cell line by mediating apoptosis, as evident by increasing cell count in necrotic stage (Fig 4).

### Nuclear condensation in breast cancer cells

**Fig 5:**
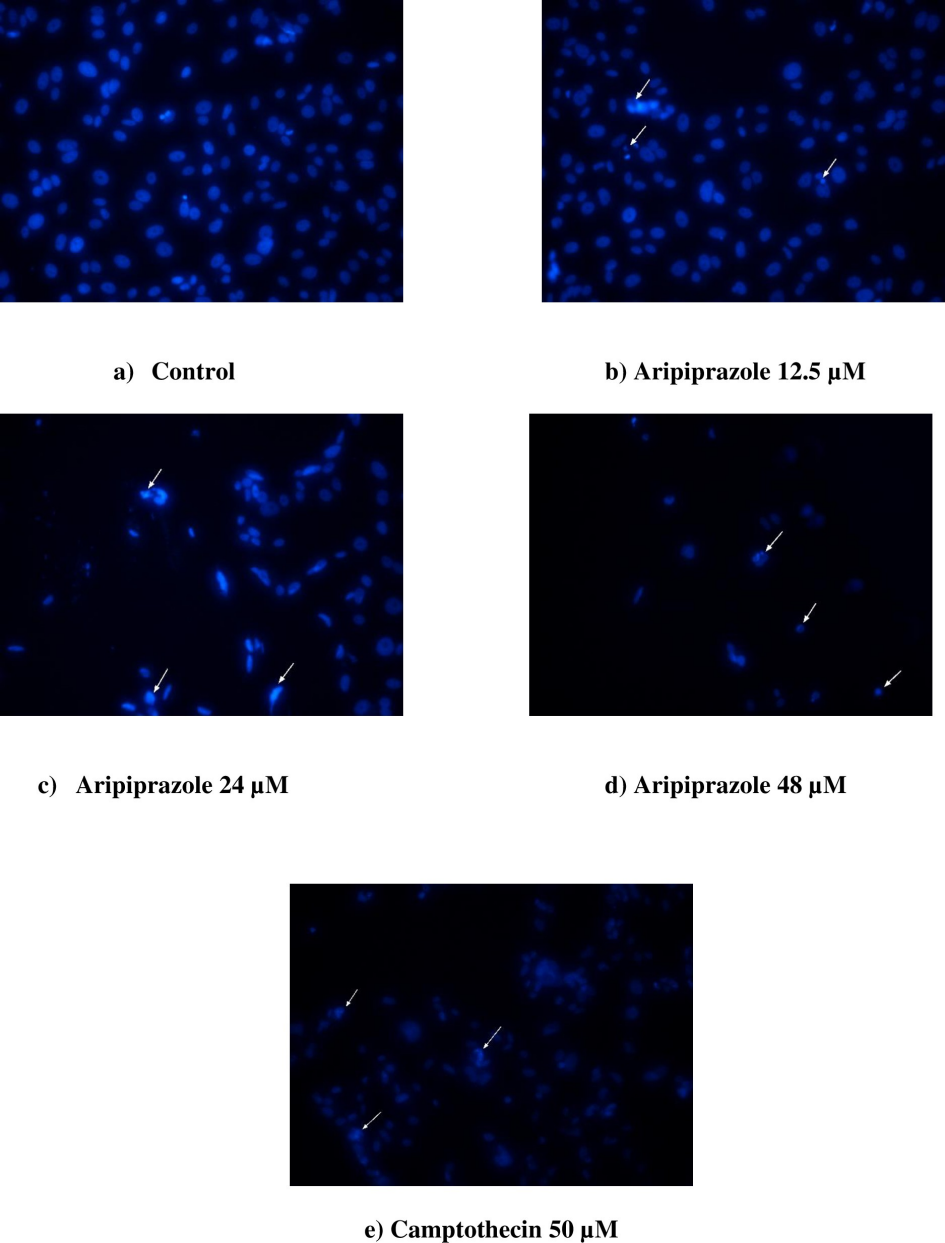
Analysis of aripiprazole-induced apoptosis in MCF-7 breast cancer cells by DAPI. a) control (untreated MCF-7), b) aripiprazole 12.5 μM, c) aripiprazole 24 μM, d) aripiprazole 48 μM, and e) camptothecin (50 μM). Images were taken at 20X magnification by using microscope TE-2000, Nikon, Japan. The nuclei were stained with DAPI (blue).

Nuclear condensation is one of the markers of apoptosis. It involves DNA fragmentation, membrane blebbing, and increase in G_1_ phase. Therefore, the effect of aripiprazole on cell was further studied by analyzing the condensation of nuclei [20]. DAPI stains binds to A-T rich region of DNA by passing through the intact cell membrane. Therefore, DAPI was used to determine nuclear condensation of the aripiprazole treated cells (12.5, 24, and 48 *μ*M) (Fig 5). Camptothecin, an anti-cancer drug, was used as a standard. Aripiprazole treated cells showed a significant nuclear condensation, and DNA fragmentation, in comparison to camptothecin (Fig 5). The cell population was also decreased.

### Aripiprazole induced subG_o_G1 breast cancer cell cycle arrest

**Fig 6:**
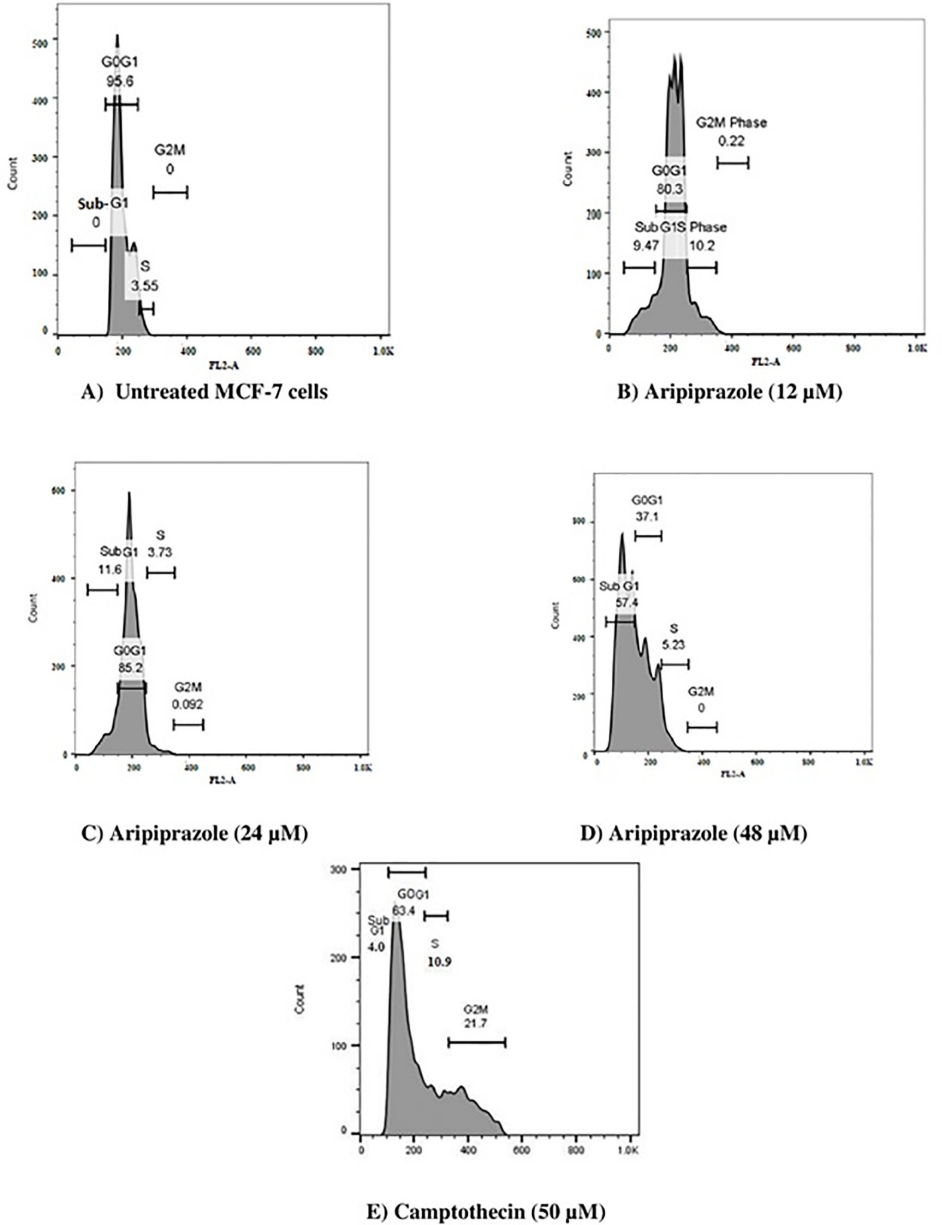
Cell cycle distribution of MCF-7 cells showing percent histogram after 48 hours of treatment. A) Untreated MCF-7 cells showing no cell population in subG_0_G_1_ phase, B) Aripiprazole (12 μM) cells showing 9.47% cell population in subG_0_G_1_, C) Aripiprazole (24 μM) cells showing 11.6% cell population in subG_0_G_1_ phase, D) Aripiprazole (48 μM) cells showing 57.4% cell population in subG_0_G_1_ phase, and E) Camptothecin (50 μM) cells showing 4% cell population in subG_0_G_1_ phase Total 10,000 cells were analyzed in triplicate during sample acquisition.

In order to further study, the mechanism of apoptosis induced by aripiprazole, cell cycle analysis was performed using PI staining methods [21]. Aripiprazole at lower concentrations *i*.*e*. 12 and 24 μM showed a significantly increased (**P<0.01) cell population in subG_0_G_1_ phase. However, at 48 μM aripiprazole, there was an increased effect and apoptotic cell population increased to 57.4% (***P<0.001) (Fig 6). These results were also significant in comparison to camptothecin that showed 4% population of subG_0_G_1_ phase cells at 50 μM.

### Altered gene expression of pro-, and anti-apoptotic genes

**Fig 7:**
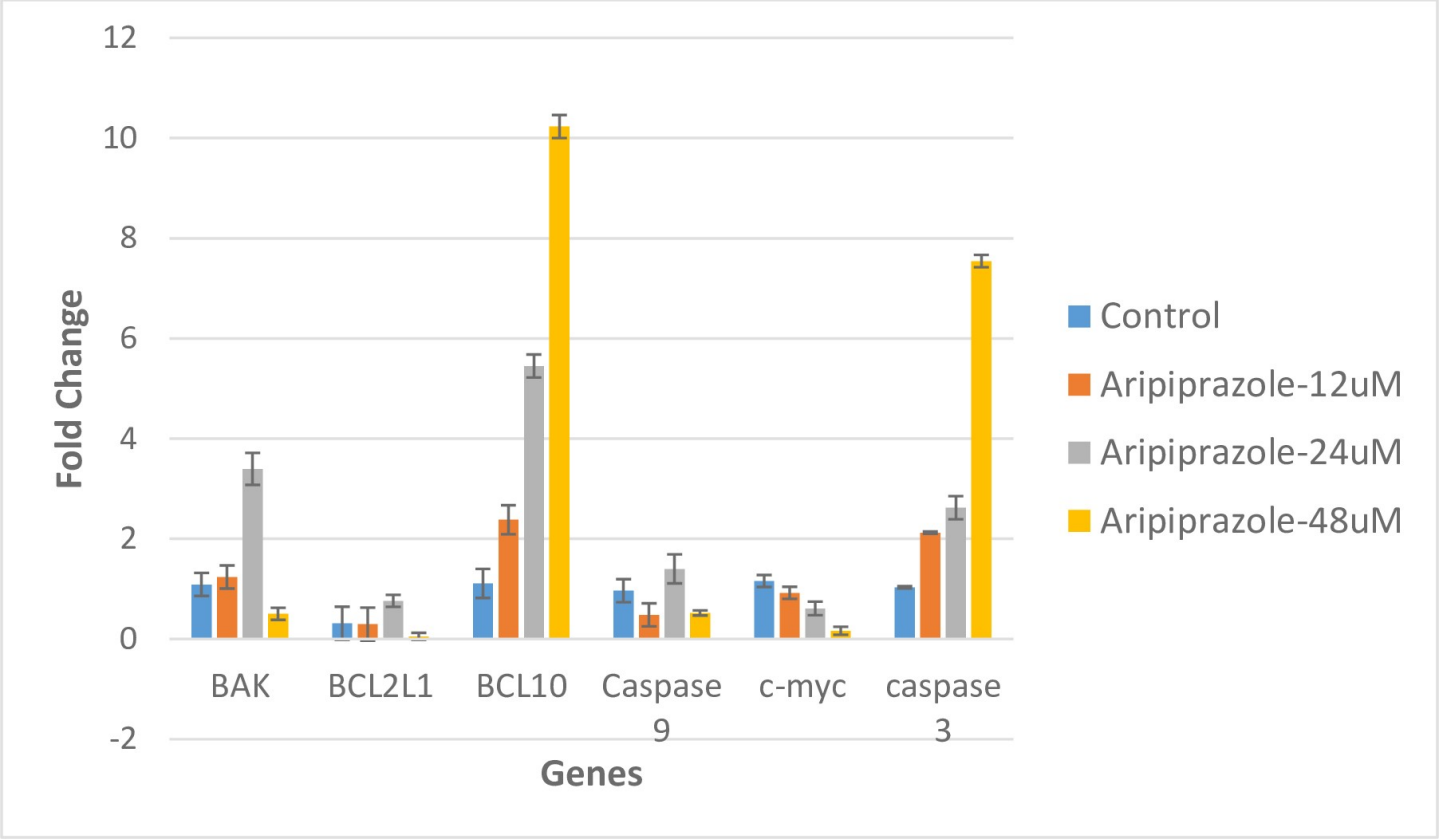
Graphical representation of fold change in mRNA expression. mRNA expression of caspase 3, caspase 9, c-myc, BAK1, BCL 1and BCL-10 calculated by comparative CT method (2^-ΔΔCT^ Method) after the treatment of aripiprazole and camptothecin. β-actin and GAPDH were used as a control gene. CT values were obtained, data was normalized against actin and fold change was calculated by Δ^CT^ method. The data are mean ± S.E.M. of three independent experiments, P-values, (***P< 0.001, ** P<0.001 and * P<0.05).

Finally, qRT-PCR was performed to evaluate the expression of certain apoptosis related genes, including caspase 3, caspase 9, c-myc, BCL-2, and BCL-10 in MCF-7 cells (Fig 7). The RNA integrity was confirmed by the intact bands in agarose gel. qRT-PCR analysis showed that aripiprazole was able to increase the expression of pro-apoptotic genes.

The expression of both the anti-apoptotic genes (BCL2L1, and c-myc) were decreased in presence of aripiprazole. The downregulation of c-myc was in dose dependent manner. Among the pro-apoptotic genes (BCL-10, BAK, and caspases 3, and 9), BCL10 and caspase 3 were upregulated in a dose dependent manner. While BAK, and caspase 9 were upregulated at 24 μM.

## Discussion

Breast cancer is the most common malignancy in women [22]. The treatment of breast cancer in early stages involves a multitude of approaches, all following an inhibition of the proliferation, and invasion of cancer cells. The strategy also employed to eliminate residual cancer, and prevent recurrence of disease [23].

A significant increase in the prevalence of breast cancer demands an urgent search of new anti-cancer drugs with better efficacy, and lower toxicity. Drug repositioning is an excellent approach to achieve this goal in relatively shorter time, in comparison to the conventional drug discovery [24]. It has several advantages, including low cost due to already available pharmacokinetics and pharmacodynamics data of drugs, and hence the method is time efficient for “bench to bed” translation [25, 26]. The current study is an example of drug repositioning in new lead discovery.

**Fig 8:**
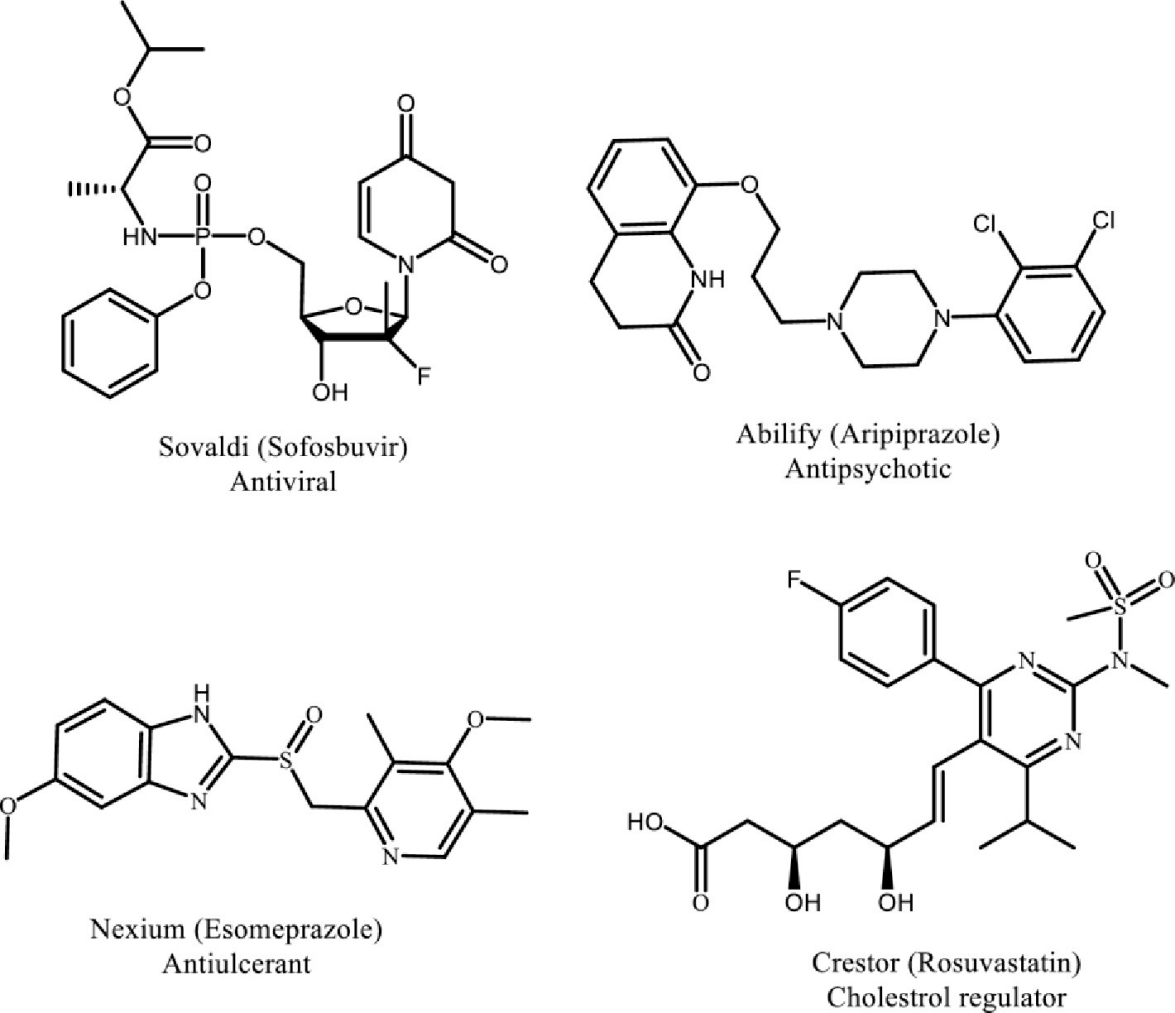
Marketed drugs with heterocyclic fragments in their structures. Different marketed drugs with heterocyclic pharmacophore that play crucial role in their physiochemical properties.

A wide range of U.S. Food and Drug Administration (US-FDA) approved drugs possess heterocyclic skeleton making them a keystone in the field of medicinal chemistry. Heterocyclic compounds and fragments play a crucial role in pharmacodynamics and pharmacokinetics properties of anticancer drugs as they enhance polarity, lipophilicity, and other physicochemical characteristics. Hence, heterocyclic moieties are present in majority of the marketed drugs [26]. For instance, among the top five US small molecule marketed drugs in 2014, four possess heterocyclic fragments in their structures (Fig 8).

Considering the above mentioned rational, we herein report the anti-cancer effects of aripiprazole, an anti-psychotic drug, along with a proposed mechanism of action. Aripiprazole was able to inhibit the proliferation in MCF-7 breast cancer cells in a concentration dependent manner. The anti-proliferative effect of aripiprazole was found to be due to the induced apoptosis, as observed in annexin V-FITC/PI analysis of aripiprazole-treated cells. An increased percentages of cell population in the early and late apoptotic stages was observed. Significant nuclear condensation was also observed in DAPI-stained nuclei of MCF-7 cells. This indicated that aripiprazole exhibit a pro-apoptotic effect in MCF-7 breast cancer cells.

Cell death can occur through different mechanisms, including autophagy, necrosis, and apoptosis. In the current study aripiprazole-treated breast cancer cells showed characteristic features of apoptosis. It is well established that apoptosis in cells can lead to morphological changes, including DNA fragmentation, cell shrinkages, alteration in membrane potential, and nuclear condensation [27, 28]. Moreover, as the major cause of cancer is alteration in the balance between pro- and anti-apoptotic factors, compounds with the ability to induce apoptosis are therapeutically important in the management of cancers. Aripiprazole, in the current study, enhanced apoptosis in breast cancer MCF-7 cells, as inferred from an increased nuclear condensation.

The cell cycle analysis showed that aripiprazole arrested the MCF-7 cells in subG_0_G_1_ phase, indicating that aripiprazole-treated cancer cells have undergone a cell death by apoptotic process. Significant increase in subG_0_G_1_ population was observed, further supporting an increase apoptosis due to aripiprazole. Interestingly, the population of subG_0_G phase has dramatically increased at a high concentration of aripiprazole. This may have prevented the cells to undergo cell division, and thus lead to cell death.

Aripiprazole was able to modulate the gene expression of certain pro- (Caspases 3, and BCL10, and anti-apoptotic genes (BCL2L1, and c-myc) those play an important role in regulating the apoptosis, and hence progression of cancer. The increased expression of pro-apoptotic genes caspases 3, and BCL10 indicated the induction of apoptosis. While expression of anti-apoptotic genes BCL2, and c-myc, were decreased in the presence of aripiprazole, which further supported the apoptotic activity of the drug.

## Conclusion

In conclusion, aripiprazole inhibits cell proliferation in breast cancer MCF-7 cells line in a concentration dependent manner. Mechanistic studies indicated that the anti-cancer effect of aripiprazole may be due to an enhanced apoptosis in MCF-7 cells line. This was further supported by observing increased nuclear condensation in MCF-7 cells line. The drug inhibited cell cycle progression in subG_0_G_1_ phase. Aripiprazole also increased the expression of certain pro-apoptotic genes (caspases 3, and BCL10), while expression of anti-apoptotic genes (BCL2L1, and c-myc) that are involved in the progression of breast cancer were decreased. This study thus indicated that aripiprazole inhibit the cancer cell proliferation through apoptosis. Further studies, however, are required to study the effect of aripiprazole on intrinsic or extrinsic pathways of apoptosis, as well as to enhance its activity by structural modifications.

## Supporting information

S1 Table Different classes of drugs screened against MCF-7 breast cancer cell line. (DOCX)

S2 Table Activity of aripiprazole in other cancer cell lines. (DOCX)
