# Supplementary material for: Antipsychotics drug aripiprazole as a lead against breast cancer cell line (MCF-7) in vitro
Source: PLoS One. 2020 Aug 3;15(8):e0235676. doi: 10.1371/journal.pone.0235676 (PMC7398703; doi:10.1371/journal.pone.0235676)
Supplement: S1 Table — (DOCX) [file pone.0235676.s001.docx]

**S1 Table:** Different classes of drugs screened against MCF-7 breast cancer cell line.

| **S/No** | **Drug Names** | **Structures** | **Mode of action** | **% Inhibition/**  **IC_50_ ± SEM^*^ (µM)** |
| --- | --- | --- | --- | --- |
| 1 | Amoxicillin trihydrate |  | β-Lactam antibiotic | 3.3% |
| 2 | Ampicillin trihydrate |  | β-Lactam antibiotic | 3.1% |
| 3 | Atorvastatin calcium trihydrate |  | Competitive inhibitor of HMG-CoA reductase | -9.7% |
| 4 | Azithromycin dihydrate |  | Macrolide antibiotics, azithromycin inhibits bacterial protein synthesis | 20% |
| 5 | Ciprofloxacin HCl |  | Broad-spectrum antibiotic of the fluoroquinolone class | 41.8% |
| 6 | Clarithromycin HCl |  | Act as bacterial protein synthesis inhibitor | 27.6% |
| 7 | Diltiazen hydrochloride |  | Muscle relaxant | 5.88% |
| 8 | Enalaprilat dihydrate |  | Inhibits angiotensin-converting enzyme (ACE) in human subjects and animals. | 53.69% |
| 9 | Glibenclamie / Glyburide |  | Inhibition of the ATP-sensitive K+ channels | 2.15% |
| 10 | Itopride hydrochloride |  | Increases acetylcholine concentrations | -1.33% |
| 11 | Levosulpiride |  | Substituted benzamide antipsychotic | 13.6% |
| 12 | Lidocaine hydrochloride monohydrate |  | Inhibiting the ionic fluxes | 12.1% |
| 13 | Linezolid |  | Synthetic antibiotic | 9.57% |
| 14 | Lisinopril dihydrate |  | Angiotensin converting enzyme (ACE) inhibtor | 71.80% |
| 15 | Ofloxacine |  | Quinolone/fluoroquinolone antibiotic | 23.6% |
| 16 | Omeprazole |  | Is a selective and irreversible proton pump inhibitor | 41.6% |
| 17 | Paracetamol (Acetaminophen) |  | Weak inhibitor of the synthesis of prostaglandins (PGs) | -1.27% |
| 18 | Piroxicam |  | Analgesic, anti-inflammatory, and antipyretic properties | 4.6% |
| 19 | Prednisolone acetate |  | Synthetic glucocorticoid | 0.12% |
| 20 | Probucol |  | Lowers the level of cholesterol in the bloodstream by increasing the rate of LDL catabolism. | -33.5% |
| 21 | Ropinirole hydrochloride |  | Non-ergoline dopamine agonist | -29.88% |
| 22 | Terbinafine hydrochloride |  | Synthetic allylamine antifungal | 19.67% |
| 23 | Tramadol hydrochloride |  | Opioid pain medication | 0.63% |
| 24 | Arippizorale |  | Antipyschotic; mediated through a combination of partial agonist activity at D2 and 5-HT1A receptors and antagonist activity at 5-HT2A receptors | 100.3%/  10.7 ± 0.2 |
| 25 | Atenolol |  | Beta blockers | -25.1% |
| 26 | Bromazepam |  | Lipophilic, long-acting benzodiazepine and with sedative, hypnotic, anxiolytic and skeletal muscle relaxant properties | -11.5% |
| 27 | Chlorthalidone |  | Indirectly increases potassium excretion via the sodium-potassium exchange mechanism | 8.68% |
| 28 | Cilostazol |  | Selective inhibitor of 3-type phosphodiesterase (PDE3) with therapeutic focus on increasing cAMP. | 8.68% |
| 29 | Diphenhydramine hydrochloride |  | First generation anti-histamine | -22.7% |
| 30 | Escitalopram oxalate |  | Antidepressant | -3.1% |
| 31 | (+-) Epinephrine |  | Alpha-adrenergic receptors | 50.5% |
| 32 | Trimethoprim |  | Binds to dihydrofolate reductase and inhibits the reduction of dihydrofolic acid (DHF) to tetrahydrofolic acid (THF) | -18.62% |
| 33 | Gemfibrozil |  | Potent lipid regulating drug | -2.7% |
| 34 | Hydrochlorothiazide |  | Diuretics | -14.2% |
| 35 | Levocetirizine dihydrochloride |  | Antihistamine | -25.24% |
| 36 | Levofloxacin Hemihydrate |  | Broad-spectrum antibiotic | 8.14% |
| 37 | Loratadine |  | Tricyclic antihistamine | 85.3%/  20.8±1.0 |
| 38 | Losartan potassium |  | Nonpeptide angiotensin II receptor antagonist with high affinity and selectivity for the AT 1 receptor. | -33.8% |
| 39 | Clopidogrel bisulfate |  | Acts by inhibiting the ADP receptor on platelet cell membranes. | 29.32% |
| 40 | Lumefantrine |  | Anti-malarial drug | 27.8% |
| 41 | Mefenamic acid |  | Anti-inflammatory pain killer | 8.04% |
| 42 | Mesterolone |  | synthetic, orally active anabolic-androgenic steroid (AAS)  -male sex hormone | 8.0% |
| 43 | Metformin Hydrochloride |  | Diabetic medication; helps control blood sugar level | -9.9% |
| 44 | Mexiletine Hydrochloride |  | anti-arrhythmic drug | 7.2% |
| 45 | Nabumetone |  | nonsteroidal anti-inflammatory drug (NSAID) | 3.6% |
| 46 | Nicotinic acid |  | Vitamin B3 | 6.8% |
| 47 | Oxaprozin |  | nonsteroidal anti-inflammatory drug (NSAID) | -7.1% |
| 48 | Penicillin G |  | Anti-biotic | -2.4% |
| 49 | Ramipril |  | ACE(angiotensin converting enzyme) inhibitor | -2.3% |
| 50 | Ticlopidine hydrochloride |  | Prevents blood clots | 1.1% |
| 51 | Valsartan |  | Angiotensin receptor blockers.  Treats high blood pressure and congestive heart failure | -7.1% |
| 52 | Tri-methyl-phloroglucinol |  | Anti-pasmodic | -14.8% |
| 53 | Cefadroxil monohydrate |  | *β*-lactam antibiotic | -10.8% |
| 54 | Cefixime trihydrate |  | Antibiotic | -1.6% |
| 55 | Ceftriaxone sodium. 3 H_2_O |  | Antibiotic (bactericidal drug) | -0.4% |
| 56 | Cefuroxime axetil |  | Antibiotic | -3.9% |
| 57 | Dextro-methorphan hydrobromide |  | dissociative anesthetic | 4.9% |
| 58 | Famotidine |  | Famotidine is used to treat and prevent ulcers | -1.0% |
| 59 | Gliclazide |  | Sulfonylureas (that are used to lower the blood sugar level) | 5.0% |
| 60 | Hydrocortisone sodium succinate |  | Treats severe allergic reactions, arthritis, blood diseases, breathing problems, certain cancers, eye diseases, intestinal disorders, and skin diseases | -13.0% |
| 61 | Labetalol hydrochloride |  | beta blockers  (used to treat high blood pressure) | 32.7% |
| 62 | Mirtazapine |  | antidepressant | 17.2% |
| 63 | Nifedipine |  | Calcium channel blockers. It is used to treat hypertension (high blood pressure) and angina (chest pain) | 9.5% |
| 64 | Clavulanic acid |  | Combined with amoxicillin and used as an antibiotic | Insoluble |
| 65 | Flavoxate hydrochloride |  | antispasmodic | -20.6% |
| 66 | Montelukast sodium |  | Anti-allergic | 28.4% |
| 67 | Sulfadoxine |  | Anti-malarial drug | -20.1% |
| 68 | Trimebutine maleate |  | It is used to treat irritable bowel syndrome (spastic colon) | 15.8% |
| 69 | Bupropion hydrochloride |  | Anti-depressant | 22.2% |
| 70 | Cinitapride |  | Treats gastrointestinal disorders | 12.7% |
| 71 | Clozapine |  | Antipsychotic medication | 35.4% |
| 72 | Domperidone |  | Used to treat nausea and vomiting | 44.1% |
| 73 | Flunarizine Dihydrochloride |  | Calcium channel blocker | 66.5%/  40.3±2.03 |
| 74 | Risperidone |  | Antipsychotic medicine | 14.0% |
| 75 | Sertraline hydrochloride |  | Treat depression, obsessive-compulsive disorder, panic disorder, anxiety disorders, post-traumatic stress disorder (PTSD), and premenstrual dysphoric disorder | 101.6%/  11.6 ± 0.1 |
| 76 | Ziprasidone hydrochloride |  | Antipsychotic medication | 43.8% |
| 77 | Beclomethasone dipropionate |  | Is used to prevent and control symptoms (wheezing and shortness of breath | 44.7% |
| 78 | Ceftazidime |  | Antibiotic | -4.8% |
| 79 | Crotamiton |  | Scabicides and antipruritics | -0.12% |
| 80 | Pioglitazone hydrochloride |  | Thiazolidinedione; increases the body's sensitivity to insulin | 2.9% |
| 81 | Ketoprofen |  | nonsteroidal anti-inflammatory drugs (NSAID) | 2.9% |
| 82 | Lincomycin hydrochloride |  | Antibiotic | -15.4% |
| 83 | Loperamide |  | Used to treat gastroenteritis, inflammatory bowel disease, and short bowel syndrome | 100.6%/  13.8±1.7 |
| 84 | Trazodone Hydrochloride |  | Antidepressenat | 21.5% |
| 85 | Triamcinolone Acetonide |  | Synthetic  corticosteroid | 45.8% |
| 86 | Venlaflaxine HCl |  | Antidepressant | 29.8% |
| 87 | Fluoxetine HCl |  | Fluoxetine is used to treat major depressive disorder | 100.3%/  14.3±0.8 |
| 88 | Ketotifen Fumarate |  | Antihistamine for eye infetcions | 13.03% |
| 89 | Pantoprazole Sodium |  | Is a proton pump inhibitor that decreases the amount of acid produced in the stomach | 27.3% |
| 90 | Pyridoxine HCl |  | (Vitamin B6) | -8.1% |
| 91 | Terbutaline sulfate |  | Used as inhaler in the management of asthma | -23.0% |
| 92 | Entecavir monohydrate |  | Oral antiviral drug | -16.5% |
| 93 | Hydroxyprogesterone caproate |  | Synthetic female hormone- to reduce the risks of preterm delivery | 94.6%  19.8 |
| 94 | Suxamethonium  HCl |  | Medication used to induce muscle relaxation, short term paralysis or to help with tracheal intubation. | 58.7%/  40.7 |
| 95 | Topiramate |  | anticonvulsant (antiepilepsy) drug | -10.1% |
| 96 | Candesartan cilexetil |  | is a drug used for treating high blood pressure (angiotensin) | -30.5% |
| 97 | Citalopram HBr |  | Antidepressant | 27.3% |
| 98 | Nitrazepam |  | hypnotic drug | -11.8% |
