# Supplementary material for: Antipsychotics drug aripiprazole as a lead against breast cancer cell line (MCF-7) in vitro
Source: PLoS One. 2020 Aug 3;15(8):e0235676. doi: 10.1371/journal.pone.0235676 (PMC7398703; doi:10.1371/journal.pone.0235676)
Supplement: S2 Table — (DOCX) [file pone.0235676.s002.docx]

**S2 Table**: Activity of Aripiprazole in other cancer cell lines.

| **Cancer Cell line** | **IC_50_ ± SEM (µM)** |  |
| --- | --- | --- |
| **MDA-MB-231**  **(Triple Negative Breast Cancer Cell Line)** | 19.83 ± 0.27 | **Doxorubicin**  0.5 ± 0.07 |
| **AU565**  **(Her2 Positive Breast Cancer Cell Line)** | 18.02 ± 0.44 | **Doxorubicin**  0.34 ± 0.02 |
| **BT-474**  **(Triple Positive Breast Cancer Cell Line)** | 36.42 ± 0.12 | **Doxorubicin**  2.2 ± 0.43 |
